# Targeting Arterial Dysfunction in Cardiovascular Disease Using Stem Cell‐Based Therapies

**DOI:** 10.1096/fj.202600649R

**Published:** 2026-03-16

**Authors:** Yun‐Yu Ma, Shu‐Yao Zhu, Yi Song

**Affiliations:** ^1^ Department of Cardiology Fuwai Yunnan Cardiovascular Hospital Kunming China; ^2^ Fuwai Yunnan Cardiovascular Hospital Affiliated Cardiovascular Hospital of Kunming Medical University Kunming China; ^3^ Department of Anesthesiology The Second Affiliated Hospital of Nanjing Medical University Nanjing China

**Keywords:** blood vessel regeneration, cellular metabolism, endothelium, phenotypic plasticity, stem cell transplantation, vascular smooth muscle cells

## Abstract

Arterial regeneration represents a critical frontier in cardiovascular medicine, as progressive endothelial dysfunction, maladaptive vascular smooth muscle cell (SMC) plasticity, and chronic inflammation drive atherosclerosis, restenosis, and vascular aging. Although current therapies such as pharmacological risk‐modifying therapies and interventional revascularization procedures mitigate the risk and delay the progression, they are still unable to restore vascular integrity. Stem cell‐based strategies were initially conceived to replace the lost vascular cells directly; however, accumulating evidence indicates their therapeutic benefits arise from paracrine mechanisms including regulation of endothelial repair, modulation of SMC phenotypic switching, and attenuation of inflammatory signaling. This paradigm shift has expanded the regenerative landscape to encompass endothelial progenitor cells, mesenchymal stromal cells, induced pluripotent stem cell‐derived vascular lineages, and engineered extracellular vesicle platforms. Parallel advances in biomaterials, mechanically tuned scaffolds, and hybrid cell‐matrix constructs provide more physiologic microenvironments for vascular repair and enhance the retention, potency, and safety of regenerative therapies. Concurrently, gene editing, metabolic reprogramming, and hypoxic preconditioning further refine the functional capacity of stem cell‐derived products, enabling targeted correction of endothelial instability and improving regulation of vascular remodeling. Integration of multi‐omic profiling and high‐resolution vascular phenotyping now positions the field to align regenerative strategies with patient‐specific determinants of disease. This review integrates current knowledge on stem cell‐mediated endothelial regeneration, SMC phenotype regulation, and bioengineered vascular interventions, and examines emerging precision‐medicine frameworks poised to guide next‐generation therapies that link mechanistic principles with translational progress to enable durable restoration of arterial structure and function, and long‐term vascular health, thereby providing a theoretical basis for future research.

## Graphical abstract

Graphical presentation of stem cell‐based therapies for arterial dysfunction, endothelial repair and regeneration strategies. SMC, smooth muscle cell.

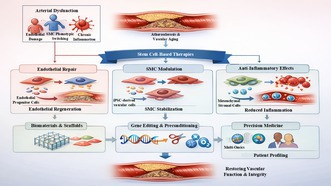

## 1 Introduction

Arterial regeneration has emerged as a central challenge in cardiovascular medicine because the adult human heart and vascular system possess very limited natural healing capacity, as cardiomyocytes in adult humans have an annual renewal rate of only approximately 0.5%, which is insufficient to repair major damage, thereby progressing to end‐stage heart failure in almost 15% of patients with heart conditions [1]. It is further driven by the rising global burden of atherosclerosis, vascular disease, and restenosis after mechanical interventions [2]. These conditions share a unifying pathology that is, progressive disruption of endothelial integrity, maladaptive SMC plasticity, and chronic inflammation that destabilize the vascular wall. Several pharmacological agents including lipid‐lowering agents such as statins (atorvastatin, rosuvastatin) and PCSK9 inhibitors (evolocumab, alirocumab); antihypertensive drugs including angiotensin‐converting enzyme inhibitors (enalapril, ramipril), angiotensin II receptor blockers (losartan, valsartan), β‐blockers (metoprolol), and calcium channel blockers (amlodipine); antithrombotic agents (aspirin, clopidogrel, ticagrelor); and anti‐inflammatory therapies such as colchicine and IL‐1β inhibition (canakinumab) along with interventional therapies such as angioplasty, stenting, and bypass surgery have been practiced. It has been shown that these interventions primarily slow the progression or mechanically restore blood flow but fail to restore arterial structure or function. Resultantly, regenerative strategies capable of reinstating endothelial homeostasis and modulating aberrant vascular remodeling are needed [3, 4].

Early efforts in vascular regeneration focused on the direct replacement of damaged cells using adult progenitors or embryonic stem cell‐derived lineages. However, the discovery that most transplanted cells do not engraft but instead act through paracrine mechanisms reframed the field [5]. This shift expanded the therapeutic landscape to include induced pluripotent stem cell (iPSC)‐derived endothelial cells (ECs), endothelial progenitor cells, and mesenchymal stromal cells, each capable of modulating inflammation, enhancing barrier repair, and influencing SMC phenotypic transitions. However, heterogeneity in cell identity, functional immaturity, and limited persistence have constrained clinical translation [6, 7]. Nevertheless, recent bioengineering advances have begun to address these limitations. Engineered extracellular vesicles, synthetic scaffolds, and mechanically tuned vascular grafts improve delivery, retention, and mechanotransduction within the arterial microenvironment [8]. In parallel, gene editing and metabolic or hypoxic preconditioning strategies enhance the regenerative potency of stem cell‐derived products, offering greater precision and reproducibility compared to earlier cell‐based interventions. These innovations point toward next‐generation approaches that are modular, controllable, and compatible with repeated administration [9].

The emergence of precision cardiovascular medicine adds a layer of opportunity. Multi‐omic profiling, cellular barcoding, and high‐resolution vascular imaging now reveal patient‐specific trajectories of ED, SMC plasticity, and inflammatory signaling [10, 11]. These insights allow regenerative interventions to be matched to the dominant biological drivers of disease, shifting the focus from generalized vascular repair to individualized modulation of arterial remodeling [12]. Having experience in cardiac, vascular, and cardiopulmonary research related to congenital heart disease and pulmonary hypertension [13, 14, 15, 16], in this review, we demonstrate current advances in stem cell‐based and stem cell‐derived strategies for arterial regeneration, with emphasis on endothelial repair, SMC phenotypic regulation, and bioengineered vascular platforms. We integrate mechanistic insights with translational progress and outline how next‐generation, precision‐guided regenerative therapies may redefine the management of arterial disease.

## 2 Arterial Biology and Regenerative Constraints in Cardiovascular Disease (CVD)

### 2.1 EC Turnover and Dysfunction

The balance between EC renewal and loss is necessary to maintain vascular integrity and function. However, disrupted turnover leads to ED, which serves as the main pathological condition of CVD. This is characterized by decreased nitric oxide (NO) availability, which acts as a precise measurement of both local and body‐wide blood vessel disorders [17]. Impaired NO signaling arises from convergent mechanisms, which include diminished endothelial nitric oxide synthase activity, altered intracellular signaling, oxidative degradation of NO, and redox imbalance within the arterial wall [18]. These alterations precede the vascular inflammation, tone dysregulation, and remodeling processes.

Further, EED are characterized by the loss of endothelial barrier integrity and a shift toward a pro‐inflammatory and pro‐coagulant cellular state. ECs upon activation attract leukocytes, inhibit natural anticoagulation and fibrinolysis and increase the production of tissue factor and von Willebrand factor which in turn break down the normal vascular homeostasis [19, 20]. Notably, ED also creates an environment throughout the body that leads to both systemic inflammation and blood clotting disorders, while the activation of ECs in specific areas of the body causes both blood clot formation and localized damage to blood vessels, thus showing how ECs in the body respond to cardiovascular diseases differently depending on their location [21, 22].

ECs of the arteries experience a specific functional stress due to high shear stress and close interaction with smooth muscle cells of blood vessels. In atherosclerosis, ED develops through the persistent endothelial activation and improper cellular signaling [23]. The classical models of mechanical injury show endothelial stress responses initiate the process of leukocyte recruitment and vascular inflammation without causing structural damage [24]. ECs release danger‐associated molecular patterns, including high‐mobility group box 1 protein and heat shock proteins, that enable monocytes to adhere while they initiate plaque formation without visible damage to the endothelial lining [24].

Irrespective of this, chronic and sterile inflammatory conditions also potentiate the ED through immune dysregulation, oxidative stress, and metabolic imbalances, thereby increasing the risk of CVD. In short, the endothelium develops regenerative limitations due to the above‐mentioned medical conditions and functions as an essential link between inflammatory processes and CVD risk, which serves as both a diagnostic indicator and a driving force behind accelerated CVD progression [25, 26].

### 2.2 Vascular Smooth Muscle Cell Plasticity

VSMCs determine all arterial remodeling processes that occur during normal body functions and CVD development. Healthy arteries maintain their vascular tone through VSMCs, which mostly display a contractile phenotype that protects their blood vessel structure [27]. Under pathological conditions such as inflammation, oxidative stress, or mechanical injury, VSMCs de‐differentiate, proliferate, and migrate. These transitions lead to silencing of contractile gene programs and development of new lineage markers which encompass macrophage‐like, osteochondrogenic, mesenchymal, and myofibroblast‐like cell types, hereby making it very difficult to trace the lineages in affected blood vessels [28].

The phenotypes are mainly driven by their connections with extracellular matrix elements and internal cellular signaling systems. The connection of the cytoskeleton with the extracellular matrix is mediated via mechanotransducers called the integrin proteins that essentially determines the fate of VSNC cells [17, 29]. Loss of integrins such as α1β1 and α7β1, which normally bind collagen IV and laminin, leads to a shift from a spindle‐shaped contractile phenotype to a synthetic state which accelerates intimal thickening. VSMCs in their synthetic state show higher integrin levels, which include α2β1 and α5β1 and αvβ3, as these integrins support extracellular matrix deposition and fibronectin assembly, while they safeguard VSMCs from apoptosis caused by oxidized low‐density lipoprotein, thus stabilizing the plaque instead of making it smaller [30].

Further, post‐transcriptional regulation establishes additional control over VSMC plasticity because microRNAs function as essential regulators of phenotypic transitions. The Dicer‐dependent pathways of microRNAs control both the ability of cells to grow and their development into contractile cells [30]. The miR‐143/145 cluster strengthens the contractile phenotype, while miR‐21, miR‐221, and miR‐222 drive VSMC cells to grow and adopt synthetic cellular behavior. The VSMC phenotype shows dynamic changes because multiple regulatory mechanisms control its expression. These regulatory layers underscore that the VSMC phenotype is not fixed but dynamically reprogrammed in response to vascular injury, which has critical effects on arterial remodeling and disease progression [31].

### 2.3 Inflammatory Milieu

Atherosclerosis is a chronic inflammatory state of the arterial wall that develops through three major processes comprising disease initiation, progression, and thrombotic complications. Under physiological conditions arterial ECs resist leukocyte adhesion while they sustain their normal state of immune system protection accompanied by the production of NO, along with maintenance of anti‐coagulant and anti‐inflammatory surface. While in pathological conditions, like atherosclerosis ECs develop a pro‐inflammatory phenotype which produces adhesion molecules and chemokines that attract leukocytes to the arterial wall as a response to metabolic stressors and cardiovascular risk factors [32]. The vascular cell adhesion molecule‐1 (VCAM‐1) functions as a key component that enables the selective binding of monocyte and T lymphocyte to ECs, and thus creates the initial cellular structure of early atherogenic lesions [33, 34].

Endothelial activation depends on two specific factors which include biochemical and biomechanical signals. The intimal layer of arteries develops inflammation due to accumulation of modified lipoproteins, which activates redox‐sensitive pathways that lead to nuclear factor‐kB activation, creating persistent VCAM‐1 expression. Pro‐inflammatory cytokines exert the boosting effect on the existing response. The process of lesion development occurs throughout different areas depending on nearby blood flow patterns [35, 36]. ECs use laminar shear stress to activate their protective mechanisms which create nitric oxide and build their defense against oxidative stress while disturbed flow conditions block these protective pathways and increase their production of inflammatory genes. The reason atherosclerosis develops in particular areas of arteries results from the combined interaction between lipid‐based signaling and flow‐based signaling mechanisms [37].

When ECs become activated, they attract monocytes, which move into the intima space where they transform into macrophages through the action of various chemokines and growth factors. Monocyte chemoattractant protein‐1 and macrophage colony‐stimulating factor serve as the principal factors that drive this process. These macrophages internalize modified lipoproteins and create foam cells that build the fatty streak while simultaneously acting as continuous sources of inflammatory mediators [38, 39]. The development of lesions progresses when T lymphocytes enter the area through chemokine gradients, which maintain their activation of macrophages. The plaque contains an intricate inflammatory network that operates through multiple cytokines and chemokines and various cellular components. The early stages of lesion development proceed through three main pathways, which include monocyte recruitment and macrophage expansion and continuous endothelial‐immune interactions [40].

The process of atherosclerosis progression leads to stable plaque formation which is susceptible to rupture. The arterial wall cytokine signaling system boosts the breakdown of extracellular matrix through macrophage‐derived matrix metalloproteinases and prevents collagen production by vascular smooth muscle cells [39], hereby making the fibrous cap more vulnerable to rupture. Inflammatory signaling activates two processes, which make plaques more likely to cause blood clots. The first process results in atherosclerosis. The second process makes plaques less stable while increasing the risk of blood clots. The arterial microenvironment becomes hostile, which prevents natural healing processes and stops body regeneration from happening [41, 42].

## 3 Stem Cell Artery Interactions: Moving Beyond Cell Replacement

### 3.1 Paracrine Signaling as the Dominant Therapeutic Mechanism

**FIGURE 1:**
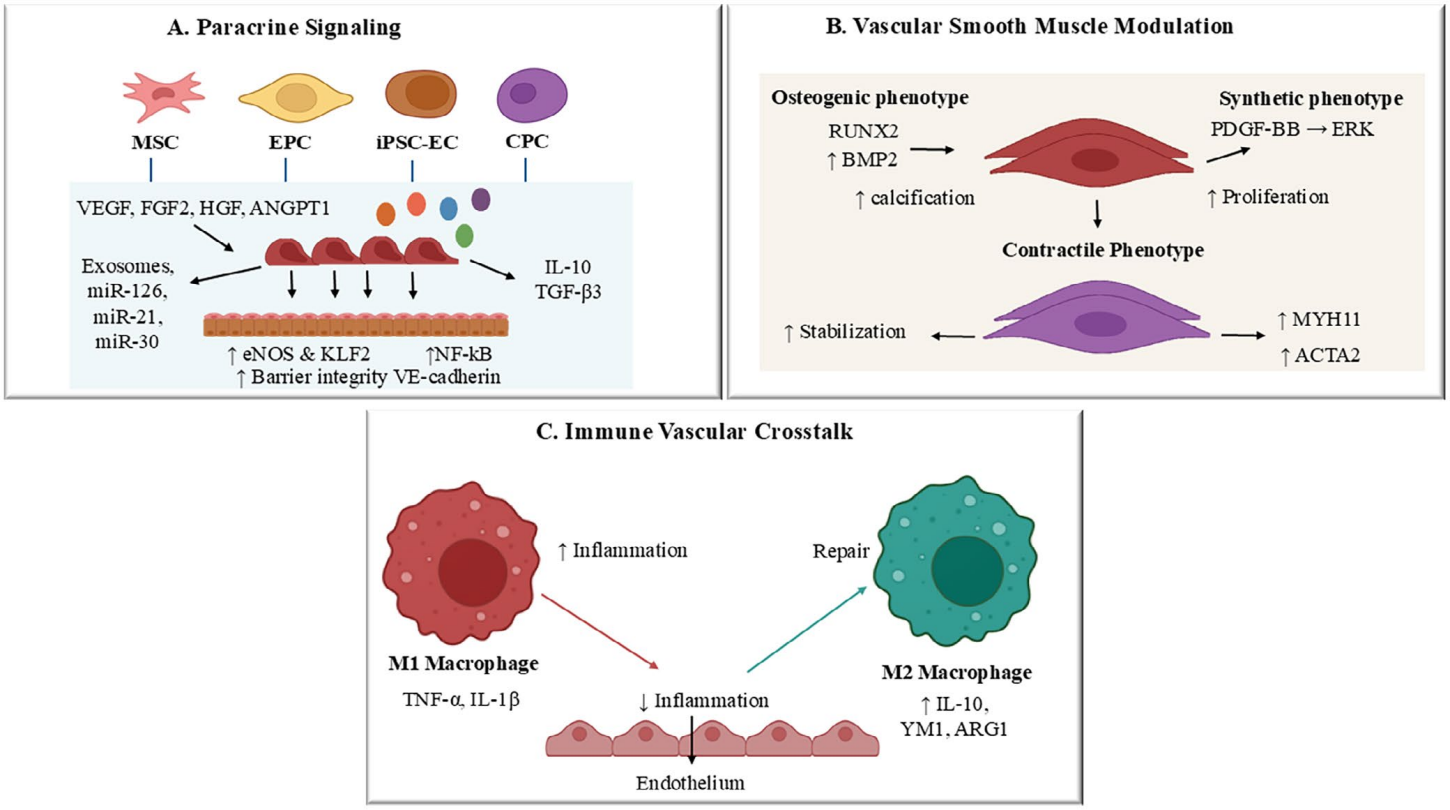
An integrated mechanism by which stem cell‐derived signals restore arterial homeostasis. (A) Paracrine signaling. The coordinated release of trophic factors and regulatory microRNAs occurs when mesenchymal stem cells, endothelial progenitor cells, and induced pluripotent stem cell–derived endothelial cells and cardiac progenitor cells secrete their exosomes. The paracrine signals lead to endothelial nitric oxide synthase and KLF2 expression increases, VE‐cadherin stabilization, and endothelial barrier function improvement while they block NF‐κB–driven inflammatory response. The simultaneous release of IL‐10 and TGF‐β3 creates an environmental condition that supports both anti‐inflammatory processes and tissue repair. (B) Vascular smooth muscle cell (VSMC) modulation. Stem‐cell‐derived mediators control the different ways VSMC cells can change their characteristics. Osteogenic signals include RUNX2 and BMP2, which lead to blood vessel calcification and plaque hardening, while PDGF‐BB activation of ERK causes VSMCs to develop a synthetic and proliferative cell type. The body repairs itself through paracrine signals that help restore contractile function by boosting MYH11 and ACTA2 levels, thus maintaining medial stability and preventing harmful structural changes. (C) Immune‐vascular crosstalk. Stem cell‐secreted modulators control macrophage polarization through their effects on M1‐induced inflammatory cytokines (TNF‐α, IL‐1β) and M2‐related tissue repair mechanisms (IL‐10, YM1, ARG1). The transition process decreases endothelial damage while increasing blood vessel restoration and decreasing long‐term vascular inflammation, representing essential factors for artery restoration.

Stem cell‐based therapies for CVD have been studied through multiple cellular platforms that have different development histories, tissue origins, and treatment purposes (Figure 1). Pleuripotent stem cells including embryonic stem cells (ESCs) possess ability to differentiate into multiple cardiovascular lineages (Table 1) [43]. Additionally, several adult stem cells including mesenchymal stem cells (MSCs), adipose‐derived stem cells (ASCs), and bone marrow‐derived mononuclear cells (BM‐MNCs) have shown the potential to repair cardiac cells. A shred of evidence highlighted the role of endothelial progenitor cells in maintaining vascular homeostasis and identified the tissue‐resident vascular progenitors that exist within arterial walls as a potential contributor of vascular repair. Although different stem cell therapies have been successively used in cardiac homeostasis, variations in scalability, immune responses, ethical issues, and clinical progress limit their long‐term efficacy as they all fail to achieve long‐term graft survival and permanent blood vessel regeneration [44, 45].

**TABLE 1:** Stem cell types and their mechanistic contributions to arterial regeneration.

Stem cell type	Primary mechanistic axis	Key molecular/Paracrine mediators	Arterial targets and cellular effects	Functional outcomes in arterial remodeling	Study
Mesenchymal stromal cells (MSCs)	Immune modulation; endothelial rescue; regulation of SMC phenotype	IL‐10, TGF‐β, HGF, VEGF, SDF‐1, EV‐borne miR‐21/miR‐126	Restores NO signaling; limits SMC dedifferentiation; reduces macrophage inflammation; enhances EPC activation	Improved vasodilation; reduced neointimal growth; fibrous cap stabilization; reduced arterial stiffness	[51]
Endothelial progenitor cells (EPCs)	Re‐endothelialization; vascular repair	eNOS, VEGF‐A, Ang‐1, pro‐endothelial microRNAs	Rapid endothelial resurfacing; suppresses thrombosis; restores junctional integrity	Faster endothelial recovery; reduced restenosis; better flow‐mediated dilation	[27]
iPSC‐Derived endothelial and smooth muscle cells	Direct vascular replacement; metabolic rewiring	KLD2/4, contractile SMC genes, antioxidant pathways	Stable endothelial layer formation; restoration of contractile SMC phenotype; reduced oxidative stress	Improved arterial compliance; enhanced plaque regression potential; reduced inflammation	[8]
Cardiac/vascular‐resident progenitors (c‐kit+, Sca‐1+, adventitial)	Local progenitor activation; niche regulation	PDGF‐BB, notch signaling, Wnt modulators, CXCL12	Controlled SMC renewal; indirect endothelial support; extracellular matrix remodeling	Stronger fibrous cap; controlled remodeling; reduced rupture risk	[15]
Bone marrow‐derived mononuclear cells	Early inflammatory regulation; transient SMC‐like states	GM‐CSF, anti‐inflammatory cytokines, platelet microparticles	Paracrine activation of resident SMCs; minimal direct differentiation; early inflammation shaping	Reduced early lesion expansion; limited long‐term structural contribution	[35]
Adipose‐derived stem cells (ASCs)	Angiogenic enhancement; immunomodulation	VEGF, bFGF, leptin‐linked signals, miR‐132	Increase microvascular perfusion; reduces macrophage inflammation; supports endothelial and SMC stability	Better perfusion; reduced ischemia‐induced arterial injury	[51]
Pericyte‐like progenitor cells	Microvascular stabilization; SMC‐like structural support	PDGFRβ pathway, Ang‐Tie2 axis	Strengthens microvascular wall; regulates endothelial permeability; promotes vessel maturation	Enhanced perfusion; protection from vascular rarefaction	[36]

A growing body of evidence shows that adult stem cell‐based treatments for ischemic cardiovascular disease work mainly through paracrine signaling instead of replacing damaged cells directly. MSCs release a wide range of cytokines and chemokines and growth factors which together change the microenvironment of damaged blood vessels and heart tissues [45]. The bioactive mediators show increased production under hypoxic stress because it represents a fundamental characteristic of ischemic tissues, which demonstrates that stem cells react to actual damage in their surroundings [46]. Moreover, tissue levels of angiogenic and cytoprotective factors also rise after administration of adult cell that is, MSC or BM‐MNCs. The factors that show increased tissue levels include vascular endothelial growth factor (VEGF), basic fibroblast growth factor, hepatocyte growth factor, insulin‐like growth factor‐1, and adrenomedullin [47]. The molecular changes in the study resulted in two outcomes, which included neovascularization development, infarct size reduction, and cardiac function enhancement. The findings establish a direct connection between stem cell‐derived secretomes and their ability to promote tissue repair. Irrespectively, the conditioned media from MSCs create pro‐survival signaling through Akt‐1 overexpression show strong cardiomyocyte protective effects [48]. The delivery of conditioned media from BM‐MNCs creates two effects include increasing capillary density and reducing ischemic damage in infarcted hearts, proving that soluble factors alone can produce therapeutic results [49, 50]. Similarly, the paracrine dominance as the primary mechanism of action is also observed in ASCs‐derived conditioned medium that reproduces the complete functional advantages of cell transplantation.

ASC‐derived paracrine signals operate beyond vascular protection and angiogenesis because they regulate post‐infraction inflammation, with fibrotic remodeling, metabolic adaptation, and endogenous repair programs. The effects show different spatial and temporal patterns because separate factors become active at various points during the healing process. The released mediators create autocrine effects that directly impact the stem cells, controlling their survival and growth and their ability to function over time. The traditional stem cell niche framework now expands through these findings because paracrine and autocrine signaling systems become essential components that control stem cell‐based arterial and heart regeneration [50, 51].

### 3.2 Immunomodulation in the Arterial Microenvironment

Stem cell therapies for CVD treatment show low success rates because they fail to produce sufficient tissue replacement through stem cell engraftment and stem cell differentiation into different cell types within damaged blood vessels [52, 53]. The research findings point toward immunomodulatory mechanisms as the main factors that produce therapeutic advantages. Diseased arteries, whether in acute ischemic injury or chronic cardiometabolic disease, are characterized by persistent sterile inflammation, which shows itself through endothelial activation and leukocyte recruitment and dystrophic tissue changes. In this context, signals derived from stem cells are said to set back the inflammatory milieu rather than repopulate vascular cell compartments [41].

MSCs show wide‐ranging capabilities to control both the innate and adaptive immune systems for arterial diseases. The MSCs exhibit their role by stopping neutrophils from activating and moving through the body, and decreasing the natural killer cells' ability to destroy cells while changing monocyte–macrophage cells to develop anti‐inflammatory characteristics. The effect of these factors depends mainly on secreted factors including cytokines and chemokines, lipid mediators, and extracellular vesicles, instead of ongoing cell presence in the vessel wall [54]. The process of immunomodulation affects the entire body because it alters immune cells present in the spleen, which deliver inflammatory cells to infected blood vessels.

Inflammation within arterial walls initiates lesion development while controlling plaque advancement, fibrous cap integrity, and blood clotting risk. Stem cell‐derived paracrine programs protect vascular systems through their ability to reduce proinflammatory signal transmission and their impact on three vascular protection mechanisms. These effects align with emerging evidence that immune cell targeting alone, which operates independently of stem cell delivery, can significantly modify vascular remodeling processes because inflammation functions as a central treatment method [24]. The research results demonstrate that stem cell therapy functions as a biological immunotherapy treatment for vascular diseases. Stem cells work as temporary controllers of arterial inflammation because they do not replace regular body parts. The new concept requires medical practitioners to change their methods of delivering treatment and determining appropriate dosage, while also explaining how stem cell treatments should be combined with specific immunomodulatory therapies used in cardiovascular treatment [11].

### 3.3 Metabolic and Epigenetic Reprogramming of Vascular Cells

**FIGURE 2:**
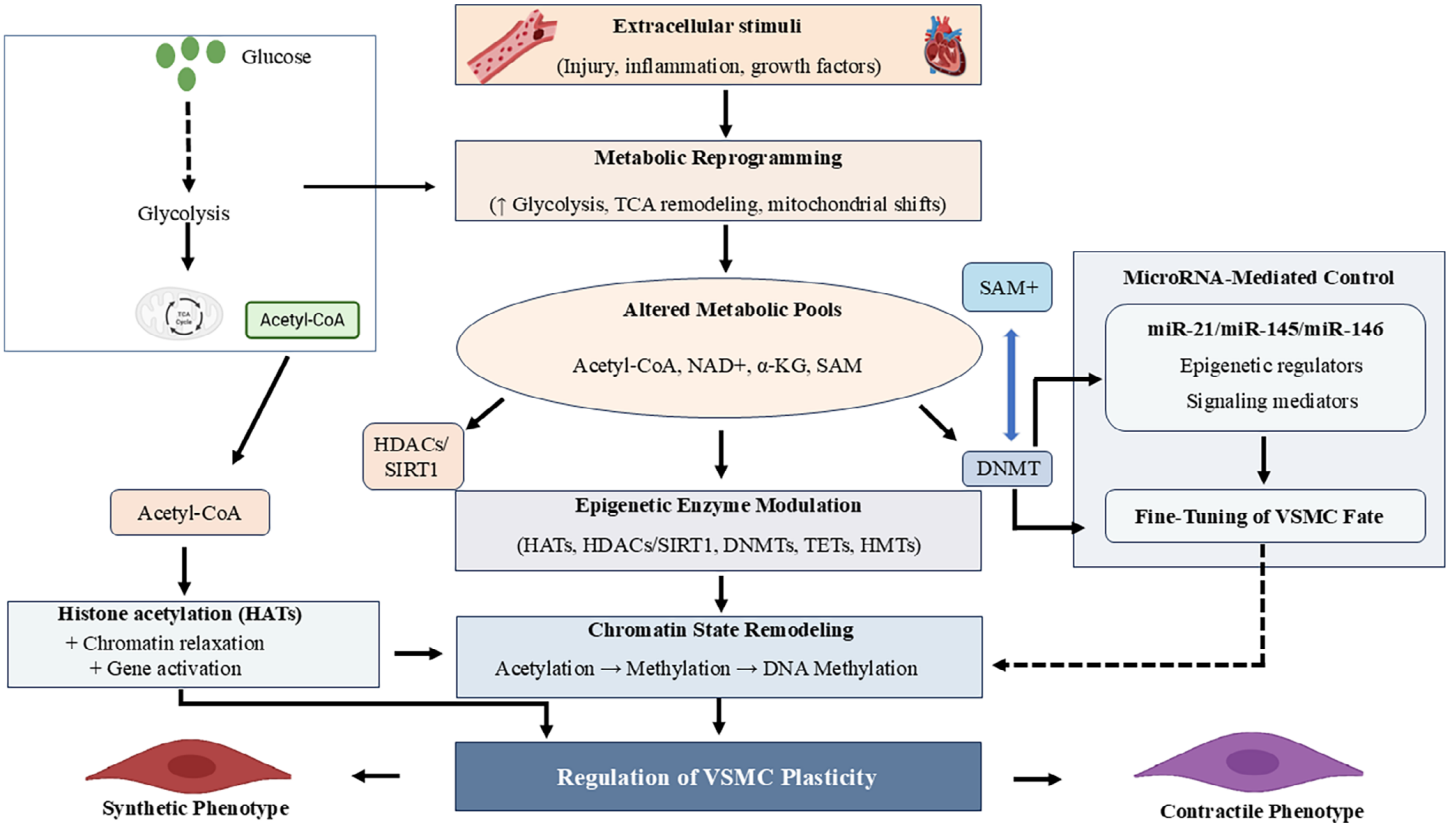
Metabolic‐epigenetic integration governing vascular smooth muscle cell (VSMC) phenotypic plasticity. Vascular smooth muscle cells (VSMCs) start metabolic reprogramming when they detect three extracellular signals included vascular injury, inflammatory mediators, and growth factors. The metabolic pathways transform to produce new metabolic compounds, including acetyl‐CoA, NAD^+^, α‐ketoglutarate, and S‐adenosylmethionine (SAM) as essential cofactors for chromatin‐modifying enzymes. The presence of acetyl‐CoA leads to increased histone acetyltransferase (HAT) activity, resulting in both chromatin relaxation and activation of genes related to synthetic phenotype development, while NAD^+^‐dependent deacetylases (HDACs/SIRT1) work to suppress this process. Metabolic intermediates also influence DNA and histone methylation through modulation of DNA methyltransferases (DNMTs) and ten‐eleven translocation enzymes (TETs) and histone methyltransferases (HMTs), which together guide chromatin structure to shift between artificial and natural states. The regulation of microRNAs through their two parallel pathways, which include miR‐21, miR‐145, and miR‐146, establishes a secondary mechanism that controls gene expression by combining metabolic and epigenetic information to determine VSMC differentiation. The three pathways, which include metabolic processes, epigenetic mechanisms, and miRNA pathways, control VSMC plasticity by determining which arterial remodeling process will dominate between proliferative/synthetic and contractile phenotypes.

Epigenetic regulation defines the fundamental characteristics of vascular cells because it controls their capacity to change between different states while determining the genetic patterns that maintain arterial health and drive disease development (Figure 2) [20, 55]. ECs and smooth muscle cells use metabolic and inflammatory signals, such as DNA methylation, histone modification, and non‐coding RNAs, to develop their cellular characteristics while their genetic blueprint remains intact. The mechanisms in cardiovascular pathology become dysfunctional, leading to endothelial dysfunction, smooth muscle cell phenotypic switching, and abnormal arterial remodeling [53, 56]. The process of establishing, erasing, and reconfiguring epigenetic states needs to be understood because it serves as the basis for developing better regenerative medical techniques that extend beyond basic cell replacement methods. The process of establishing, erasing, and reconfiguring epigenetic states needs to be studied because this knowledge will help scientists develop better regenerative medical techniques that go beyond basic cell replacement methods [5, 22, 57].

Research in stem cell biology and cellular reprogramming has shown that lineage identity exists as an epigenetic limitation that can be changed. Scientists must conduct major chromatin changes, involving both promoter demethylation and the rearrangement of histone marks that control gene expression, before they can achieve pluripotency or other somatic identities. The originally defined principles for developmental and cardiac systems establish similar principles for vascular cells, which display disease‐related characteristics that result from permanent yet reversible epigenetic changes [27, 32]. The experimental research showed that DNA methyltransferases and histone deacetylases could be manipulated to achieve complete restoration of vascular gene programs, which resulted in the recovery of contractile smooth muscle characteristics and EC stability during disease states [30].

Non‐coding RNAs, particularly microRNAs, function as powerful epigenetic regulators that establish connections between metabolic stress and the behavioral patterns of vascular cells. The multiple miRNAs that target both transcription factors and chromatin modifiers and signaling intermediates enable scientists to control complex phenotypic transitions [49]. The specific miRNA networks that control smooth muscle cell plasticity with neointima development show their importance for atherosclerosis and restenosis. The effects demonstrate how multiple regulatory factors produce better results than single‐factor treatments combined to create broader reprogramming systems. The study demonstrates that stem cell signals control arterial biology through two main mechanisms, which operate through epigenetic changes and metabolic pathway alterations [42, 55]. The regenerative therapies do not create new cell identities but instead restore vascular cells to their original state through chromatin modifications and transcriptional memory changes. The approach targets epigenetic regulators through two methods which include stem cell‐based techniques. The approach operates as an effective method to restore damaged arteries through permanent restoration techniques [39, 45].

## 4 Endothelial Regeneration and Functional Re‐Endothelialization

### 4.1 Endothelial Progenitor Cells and Vascular Repair

Current research evidence demonstrates that blood vessel restoration after endothelial damage occurs because resident ECs perform the main role in the process, while circulating endothelial progenitor cells (EPCs) establish a permanent presence in the body [58]. Lineage tracing together with transplantation research show that ECs from nearby blood vessels start to divide and create new blood vessels through the process of re‐endothelialization. However, circulating EPCs only produce minor effects on blood vessel restoration across various types of vascular systems which include both major arteries and small specialized blood vessel networks. Earlier constructs that structure EPC‐engraftment were challenged by these observations, and they put vascular repair back into a tissue‐driven process of regeneration [29, 59].

The ECs in the body show different functional abilities and form a hierarchical system which determines their ability to multiply but their most regenerative cell types remain unknown because their specific identification markers have not yet been established [59]. The discovery of small groups of ECs that possess high cloning ability and self‐renewal function proves the existence of endothelial stem‐like cells in adult tissues. The transcriptional control of EC‐cycle re‐entry functions through tissue‐specific mechanisms that depend on local angiocrine signals and transcription factors for its operation. Regenerative signaling pathways determine how ECs will repair themselves through their dual ability to either promote endothelial repair or lead to fibrosis development [60, 61].

The process of vascular repair needs more than just the activation of existing cells because it requires the use of external EC sources like from pluripotent stem cells and through direct lineage reprogramming. The use of induced pluripotent stem cell‐derived ECs for re‐endothelialization after simulated injuries in experimental models shows their potential but their clinical application remains limited because of three main issues which include time requirements and safety issues and difficulties in scaling up their use [60, 62]. These restrictions made researchers develop direct transdifferentiation methods which enable the transformation of somatic cells through direct conversion into endothelial‐like cells. The cells demonstrate strong ability to multiply while maintaining their endothelial characteristics and establishing blood vessel connections in living organisms which shows their potential for medical use [45, 63].

It has been reported that circulating EPCs and ECs can promote vascular repair through their paracrine signaling activity despite their limited ability to integrate into the vascular system. The research demonstrated that conditioned media with soluble angiocrine factors and endothelial‐derived macrovesicles were capable of inducing ECs to grow and move while maintaining their life functions [21, 23]. Endothelial microparticles transfer bioactive RNA molecules which include microRNAs to recipient cells. This process leads to the activation of angiogenic signaling pathways which include Ras‐MAPK. The observations support a central principle of vascular regeneration which states that endothelial repair depends more on the signaling pathways that transform existing ECs than on the process of replacing damaged ECs and that EPCs primarily function to control the healing environment in the body [43, 64].

### 4.2 iPSC‐Derived ECs

iPSCs serve as a reliable system to generate ECs that possess specific arterial or venous characteristics while using serum‐independent chemical testing methods. Recent research shows that developmental signaling pathways, especially VEGF pathways, can be used to create arteriovenous specifications without the need for multiple complex procedures [20]. The new differentiation method, which uses VEGF signaling control, produces endothelial precursor cells that transform them into arterial‐like or venous‐like ECs through controlled and reproducible processes, which provide better results than existing methods that require multiple pathways to be modified at the same time. This massive cytological expansion demonstrates the applicability of these approaches with special interest in vascular engineering or regenerative applications such as ECM assaying and pathway analysis [59, 65].

iPSC‐derived arterial and venous ECs functionally replicate essential lineage‐specific characteristics like somatic endothelium. Venous‐like ECs show increased leukocyte adhesion because they produce more adhesion molecules like E‐selectin. Arterial‐like ECs show different barrier responses which result from vasoactive stimuli because they maintain extended transendothelial electrical resistance decreases after thrombin exposure [40, 59]. The two cell types show decreased inflammatory responsiveness when compared to primary ECs which show strong ICAM‐1 induction but only small increase in E‐selectin and VCAM‐1 production after exposure to pro‐inflammatory cytokines. The ESC‐derived ECs show this immature inflammatory phenotype because they have not yet completed their final development stage which allows them to maintain lower levels of host inflammation during cell transplantation [9, 53]. The endothelial function assessment shows that iPSC‐derived ECs display positive interactions with biomaterial scaffolds which are used for vascular reconstruction purposes. The cells establish adhesion to surfaces of nanofilms which possess mechanical properties that match those of native blood vessels and they proceed to multiply until they create complete monolayers while their endothelial junctions and blood compatibility remains intact. The iPSC‐derived ECs and their corresponding nanomaterials demonstrate blood‐contact safety because they do not trigger platelet activation or pro‐thrombotic signaling responses [23, 53, 56].

### 4.3 MSCs and Endothelial Support

ED serves as the main characteristic that distinguishes both ischemic and non‐ischemic cardiomyopathies because it leads to decreased function of EPCs and results in defective flow‐mediated vasodilation and excessive VEGF levels in the bloodstream. The ability of EPCs to form colonies has been associated with both negative factors and a treatment substitute according to clinical and experimental evidence [3, 44]. The field of mesenchymal stem cell research has established MSCs as effective enhancers of EC balance. The MSC administration restores endothelial function through two mechanisms which include increasing EPC numbers and improving their quality while it normalizes vasodilatory responses and blocks VEGF signaling. The treatment brings back essential endothelial functions which are lost during CVD development [7].

Allogeneic MSCs provide better endothelial support than autologous MSCs because they are genetically different from the patient who receives them. The benefits of this treatment show that the cellular fitness of the system remains intact because aging and chronic diseases restrict stem cell abilities to create new blood vessels and move through space and resist oxidative damage and support their biological environment [45, 51]. Donor‐derived MSCs from young healthy donors maintain strong paracrine functions and better activate natural endothelial progenitor cell release, while their autologous counterparts face treatment limitations due to inflammatory and metabolic stress. The observed results give scientific evidence which explains why “off‐the‐shelf” allogeneic MSC methods work and this evidence helps to solve the problem of inconsistent results which appeared in previous cell therapy studies [4, 11].

The primary method through which MSCs restore endothelial function operates through indirect pathways. The MSCs that doctors deliver to the heart use paracrine signaling to create body wide effects while they do not establish permanent roots in the body [54]. Body produces anti‐inflammatory cytokines and growth factors and chemokines, which create bone marrow niches that support EPC movement and enhance blood vessel function. The mechanism of action shows that MSC therapy functions as a treatment that supports blood vessel function instead of treating diseases through cell replacement [39].

## 5 Stem Cells in Arterial Remodeling and Atherosclerosis

### 5.1 Plaque Stability Versus Plaque Regression

The process of arterial remodeling depends on the active process of VSMCs which maintain their normal behavior until they start to build up in the intima during plaque development and healing. VSMCs' proliferation and migration create luminal narrowing in blood vessels, but these cells protect blood vessels through their formation of fibrous caps that maintain the stability of atherosclerotic plaques [66]. It has been reported that VSMCs which lose their cap‐forming ability or perform abnormally lead to increased cap formation and higher risk of plaque rupture which results from distinct mechanisms of plaque burden and plaque stability. Therapeutic strategies need to differentiate between two treatment goals which aim to suppress undesired intimal hyperplasia while they defend essential VSMC populations to create vascular stability [67, 68].

The initial excitement about BM‐MNCsrestoring plaque VSMCs through their circulation has been reduced by comprehensive research studies on cell ancestry. The research evidence shows that in chronic atherosclerosis the fibrous cap VSMCs develop mainly from cells already present in the arteries instead of from incoming progenitor cells [69]. BM‐MNCs primarily interact with the lesion microenvironment through their two main pathways which involve both inflammatory response and paracrine signaling to create permanent smooth muscle replacement. These results change the existing scientific understanding because stem cells do not primarily drive plaque regression through cellular integration but rather through their ability to influence natural body healing processes [70, 71].

The use of stem cell therapies will have a greater effect on plaque stability according to their translational research potential than their ability to reverse existing plaques. Stem cell‐derived signals will enhance fibrous cap protection by two mechanisms: their ability to decrease inflammation and control matrix degradation while also preserving the survival of vascular smooth muscle cells [72]. The distinction between these two facts matters because it affects both the assessment of treatment results and the development of future therapies needed to prevent acute thrombotic events through methods that go beyond simple plaque reduction [73].

### 5.2 Regulation of VSMC Phenotypic Switching

**FIGURE 3:**
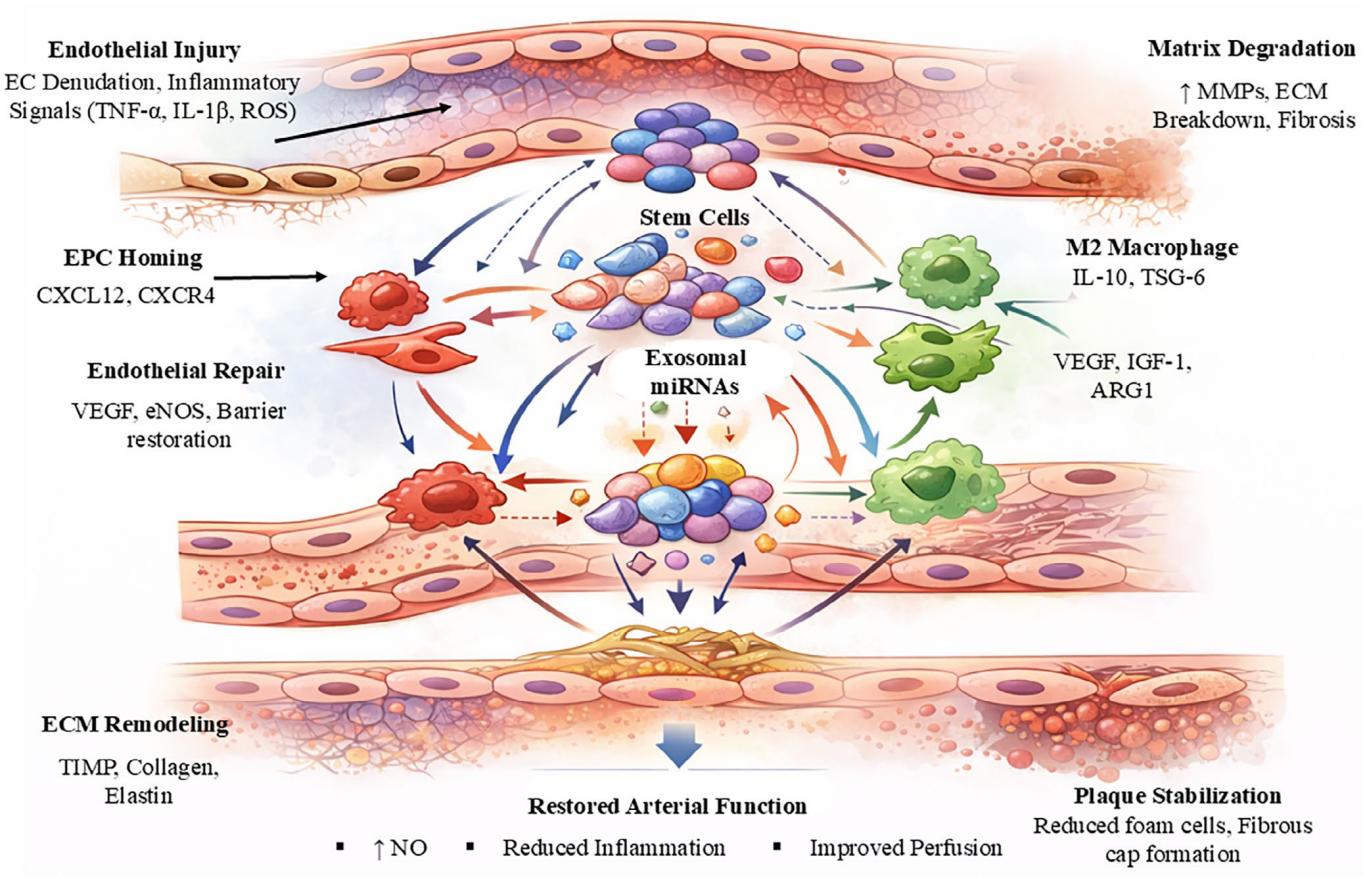
Stem cell‐mediated vascular repair through immunomodulation, endothelial regeneration, and extracellular matrix remodeling. The process begins with endothelial injury which results in the loss of endothelial cells while inflammatory signals such as TNF‐α and IL‐1β and reactive oxygen species activate the vascular system to develop dysfunction which leads to plaque destabilization. The damaged endothelium utilizes CXCL12–CXCR4 signaling to attract endothelial progenitor cells (EPCs) while simultaneously bringing stem cells to areas where blood vessels have been harmed. Stem cells create paracrine signals through their exosomal microRNA release which controls endothelial restoration and immune system defense and extracellular matrix (ECM) processing. The exosomal signaling pathway helps restore endothelial function because it enables the recovery of barrier protection and the production of nitric oxide (NO) through pathways that depend on VEGF and eNOS. Stem cell‐derived signals guide macrophages to develop into an M2 anti‐inflammatory phenotype which produces IL‐10 TSG‐6 VEGF IGF‐1 and ARG1 to decrease inflammation while protecting the matrix from destruction. The process of ECM remodeling through MMP activity control and TIMP expression increase leads to collagen and elastin reorganization together with fibrous cap development. The combination of these processes leads to atherosclerotic plaque stabilization which results in decreased foam cell buildup and restored arterial function while improving blood flow and reducing inflammatory response and maintaining vascular health.

VSMCs demonstrate exceptional ability to change their behavior when they move from their inactive state to their active state after blood vessel damage. The process of phenotypic conversion enables both rebuilding after injuries and the development of abnormal tissue structures [74]. Synthetic VSMCs downregulate canonical markers such as α‐smooth muscle actin and smooth muscle myosin heavy chain while increasing extracellular matrix production and migratory capacity. The balance between these states determines whether remodeling culminates in stable repair or progressive stenosis (Figure 3) [75].

Stem and progenitor cells operate their primary functions by using indirect methods to change the VSMC cell characteristics. The BM‐MNCswith other blood cell types establish a temporary presence at sites of injury which they use to release cytokines and growth factors that activate the resident VSMCs [76]. Although these cells may express smooth muscle markers for a short time period, most evidence supports the interpretation that such cells represent inflammatory intermediates that originate from macrophages instead of authentic terminally differentiated VSMCs. Researchers now recognize that the early studies which showed transdifferentiation evidence actually resulted from two different phenomena: marker promiscuity and phagocytosis of smooth muscle debris and cell fusion events [58, 77].

The severity and context of vascular injury determine how much circulating cells contribute to tissue remodeling. The acute high‐grade injury which results in wire‐induced neointima formation shows a high degree of inflammatory cell influx together with maximum phenotypic overlap. The process of neointimal vascular smooth muscle cell development from local arterial sources begins when inflammation reaches its conclusion. The results show that the stem cell‐based control of VSMC behavior works by controlling how resident cells change their physical appearance instead of replacing their entire cellular lineage [10, 45, 78].

### 5.3 Impact on Arterial Stiffness and Vascular Aging

Arterial stiffness shows the total structural and cellular changes that have happened in vascular walls and serves as a primary indication of vascular aging. The process is influenced by VSMCs, which change their physical characteristics to build new extracellular matrix materials and increase collagen production while destroying elastic lamellae structures [18]. The immune system brings about permanent arterial damage through its activities because it leads to the ongoing production of vascular damage at the tissue level [31, 48].

Stem and progenitor cells maintain their influence on arterial stiffness through their control of the inflammatory and healing environment. BM‐MNCsfunction as non‐permanent structural elements that control the distribution of cytokines and growth factors, determine how matrix remodeling occurs, and how vascular smooth muscle cells develop [47, 54]. Unresolved inflammation leads to continuous synthetic VSMC activation together with excessive matrix production and progressive arterial constriction. The paracrine signals that stem cells release to end inflammation actually maintain arterial flexibility while slowing down the aging of blood vessels. The research demonstrates that stem cell‐based methods function as systems that control the process of vascular aging instead of serving as tools that replace blood vessel structures. The research demonstrates that stem cell‐based methods function as systems that control the process of vascular aging instead of serving as tools that replace blood vessel structures [47, 54]. The research team aims to investigate the signaling networks that control VSMC plasticity, and they study matrix homeostasis and the process of inflammatory resolution. The methods establish a connection to developing precision medicine frameworks that use stem cells and their derivatives to restore arterial biological functions instead of reconstructing vascular structures [17, 22, 47, 54, 68].

## 6 Translational Progress

The first use of skeletal myoblasts for human heart implantation during coronary artery bypass surgery which took place exactly 20 years ago marked the beginning of cardiac cell therapy for clinical use. The central premise of myocardial remuscularization has not been achieved despite extensive research work includes both experimental studies and clinical trials that have been conducted since that milestone [79]. The results of clinical studies demonstrate that different adult cell types and delivery methods and dosing techniques lead to neutral outcomes which produce minor effects except for specific hard endpoints which include infarct size, ventricular function, and survival rates. These results require scientists to completely rethink the mechanism because it shows that researchers should focus on indirect methods which modify diseases instead of using cell replacement methods [57].

Meta‐analyses and early‐phase trials show that adult cell therapies have safe applications which produce minimal immune responses yet their effectiveness remains unproven because the studies lack enough strength to demonstrate it. The research studies establish this common finding which demonstrates that functional advantages emerge only if there is active myocardial cell integration into the tissues and the development of heart muscle cells [80]. The present system shows that externally introduced cells primarily operate through their ability to create chemical signals and modify immune responses instead of establishing direct connections with body tissues. The discovery that heart failure functions as a permanent inflammatory disease helps explain these results because most tested cell groups show built‐in abilities to reduce inflammation and prevent scarring [80, 81].

The research community has conducted extensive studies on two adult stem cell platforms include cardiac c‐kit‐positive progenitor cells and bone marrow‐derived mesenchymal stromal cells. Research using animal studies shows that neither CPCs nor MSCs can convert into cardiomyocytes at levels that can be used for practical purposes [81]. The two cell types work to change the microenvironment that occurs after an injury through three specific actions include reducing inflammation and fibrosis, stopping cell death, and supporting the development of new blood vessels. These effects lead to better cardiac stability because they do not lead to physical regeneration, which changes the definition of “repair” to mean that both blood vessels and immune systems must be restored to their original state [82, 83].

Clinical translation of these concepts has been demonstrated through recent randomized trials which evaluated function and scar burden; the selected clinical endpoints showed measurable improvements through heart failure‐related hospitalizations and patients‐reported quality of life assessment. Combination therapy delivered its strongest results which showed that different biological processes worked together instead of functioning independently. Research results support a model that shows that treatment advantages result from simultaneous control of three processes: endothelial function, immune system activity, and myocardial stress responses (Table 2) [2, 84]. The absence of structural myocardial regeneration, despite favorable clinical signals, underscores the need to recalibrate expectations and trial design. The medical condition of heart failure shows that single‐dose treatments will not produce enduring advantages for patients. This development has created new research interest in methods of administering multiple doses through less invasive delivery systems, which include intravenous administration allows peripheral cell sequestration to produce systemic immunomodulation [84]. The current research efforts work to isolate cell‐derived products include extracellular vesicles and secretomes, to establish more reliable and scalable results with precise mechanisms. The research shows that adult stem cells from translational studies. They can change disease progression without developing new heart muscle tissue. The medical benefits of this treatment probably stem from its ability to maintain blood vessel and immune system balance instead of restoring heart muscle function [45]. Future progress will depend on two specific conditions because scientists must achieve a mechanistic understanding while developing proper testing methods and testing bioengineered cell products through precision approaches that match treatment methods to specific disease stages. Stem cell‐guided cardiac repair has developed into a scientific method that uses biological knowledge to create vascular and immune system solutions for heart regeneration.

**TABLE 2:** Clinical trials targeting arterial outcomes with stem‐cell‐based interventions.

Stem cell type/product	Trial/phase	Patient population	Arterial endpoint assessed	Key outcomes
Allogeneic MSCs	DREAM‐HF (Phase III)	Ischemic & non‐ischemic HF	Flow‐mediated dilation (FMD); circulating EPCs; peripheral arterial perfusion indices	↑ FMD, ↑EPC colony‐forming activity; ↓ VEGF excess; reduced HF‐related events
Autologous MSCs	CONCERT‐HF (Phase II)	Chronic ischemic cardiomyopathy	Peripheral endothelial function (brachial FMD), NT‐proBNP (vascular load surrogate)	Safe but modest effects; no major change in LVEF; MSC‐treated patients showed better QoL and fewer HF hospitalizations
iPSC‐derived endothelial cells (iPSC‐ECs)	NCT03164184 (Pilot)	Critical limb ischemia	Limb perfusion (ABI), ulcer healing, arterial Doppler flow	Improved microvascular perfusion and pain score; no major safety concerns
CD34+ endothelial progenitors	ACT34‐CLI (Phase II/III)	Critical limb ischemia	ABI, rest pain, ulcer healing, amputation‐free survival	Improved amputation‐free survival and perfusion; durable microvascular benefit
Bone‐marrow mononuclear cells (BM‐MNSc)	REPAIR‐AMI (Phase II/III)	Acute myocardial infarction	Coronary flow reserve (CFR); endothelial‐dependent vasoreactivity	Improved CFR in subset; modest effects on LV recovery
Adipose‐derived regenerative cells (ADRCs)	ATHENA (Phase III)	Refractory angina	Coronary perfusion (SPECT), endothelial‐dependent perfusion reserve	Trend toward improved perfusion; limited by safety concerns
Peripheral blood EPCs	NO‐EPC trial (Phase I/II)	Pulmonary arterial hypertension	Pulmonary arterial pressure, 6MWT, endothelial markers	↓ Pulm, arterial resistance; ↑ exercise capacity
Cardiosphere‐derived cells (CDCs)	ALLSTAR (Phase II)	Post‐MI ventricular remodeling	Coronary flow reserve; arterial stiffness surrogates	Trial halted early; no major clinical benefit
Umbilical cord‐derived MSCs	RIMECARD (Phase I/II)	Ischemic HF	Endothelial function, vascular inflammatory markers	Improved endothelial function, reduced TNF‐α/IL‐6
MSC‐derived extracellular vesicles (MSC‐EVs)	NCT‐4327635 (Pilot)	COVID‐related endothelial injury	Endothelial injury biomarkers (VCAM‐1, ICAM‐1), arterial stiffness	Early signals of reduced endothelial injury markers

## 7 Bioengineering and Next‐Generation Stem Cell Strategies

### 7.1 Cell‐Free Therapies

**FIGURE 4:**
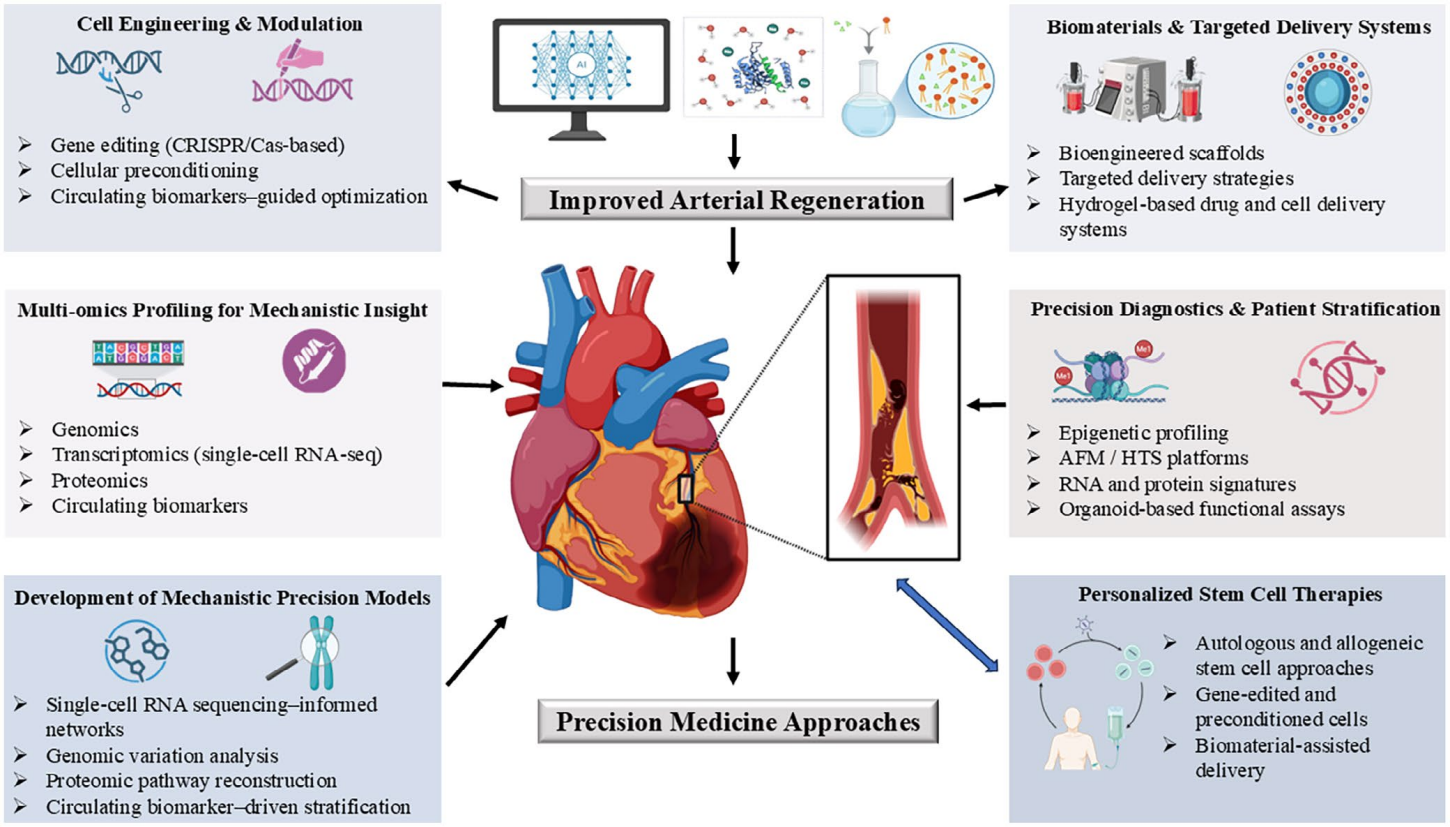
Precision medicine‐driven next‐generation stem cell strategies for arterial regeneration. Stem cell‐based therapies for arterial repair now undergo transformation through precision medicine methods that use multi‐omics profiling, mechanistic modeling, and biomaterial‐guided delivery systems. Stem cell functionality and reparative capacity develop through cell engineering techniques and modulation strategies that include CRISPR/Cas‐based gene editing and cellular preconditioning and optimization through circulating biomarker identification. Multi‐omics profiling academic research provides researchers with detailed biological understanding through its use of genomics and single‐cell transcriptomics and proteomics and circulating biomarkers to study vascular cell states and disease‐specific pathways. The research data create mechanistic precision models through single‐cell RNA sequencing networks and genomic variation analysis and proteomic pathway reconstruction and biomarker‐driven stratification which allow researchers to predict how therapies will work. The research studies multiple biomaterials together with their targeted delivery systems which include bioengineered scaffolds and hydrogel‐based drug and cell delivery platforms and targeted delivery strategies to enhance cell retention and survival and spatial control at vascular injury sites. Precision diagnostics and patient stratification approaches, including epigenetic profiling and AFM/HTS platforms and RNA and protein signatures and organoid‐based functional assays, enable individualized treatment selection. The integrated strategies work together to enhance arterial regeneration and develop personalized stem cell therapies which lead to better and individualized arterial repair methods.

The limited ability of transplanted stem cells to survive and their low rate of integration into new environments have led scientists to choose cell‐free treatments that replicate stem cell signaling activities without needing permanent cell integration (Figure 4). Currently, extracellular vehicles (EVs) included exosomes and macrovesicles, serve as the most developed method for these solutions [85]. The extracellular vesicles stem from mesenchymal stromal cells and endothelial progenitors, and iPSC‐derived vascular cells contain a specific collection of microRNAs, proteins, and metabolites that directly influence endothelial repair and decrease inflammatory response and limit the abnormal changes in SMCs behavior. Because of their nanoscale size, they can drive through the tunneled regions of the altered arterial beds, places where stem cells hardly settle [63, 86].

The operational mechanisms of EVs provide multiple advantages that benefit the treatment of arterial diseases. The cells demonstrate multiple protective effects by which they stop EC death, restore their protective functions, increase nitric oxide availability, and decrease NF‐kB pathway‐activated inflammatory responses [63, 87]. The EV cargo, which includes miR‐126 and miR‐210 and the miR‐143/145 clusters, function to inhibit SMC phenotypic switching, which results in stabilized plaques and restricted neointimal growth. The distribution of EVs throughout the body shows a preference for the spleen and liver, together with the blood circulation system, allowing these vesicles to control immune responses throughout the entire body in a manner that resembles stem cell treatment but offers better safety and consistent results [88, 89].

The arterial regeneration process is receiving new modular treatment options through the development of vesicle‐based secretome therapeutics, conditioned media therapies, engineered cytokine mixtures, and purified paracrine factors [90]. Platforms enable precise control over both dosage and release timing while maintaining consistent performance across different donors, which is a common challenge in cell therapy. The combination of EV biology with secretome engineering establishes cell‐free therapeutics as a fundamental component for advanced arterial repair methods [91].

### 7.2 Biomaterial and Vascular Scaffolds

Bioengineering advancements have developed vascular scaffolds that serve as active systems control arterial regeneration instead of acting as static support structures. Contemporary materials, together with electrospun nanofibers, decellularized extracellular matrix, and tunable hydrogels, create microenvironments that replicate arterial stiffness, topographical features, and biochemical signals [71, 92]. The scaffolds create endothelial alignment by presenting adhesive ligands and mechanotransducive signals that also support barrier maturation and restrict maladaptive SMC proliferation. The design process now uses single‐cell and spatial transcriptomics data to match scaffold properties with arterial bed molecular requirements [93].

The scaffold effectiveness has grown because stem‐cell technology integration has been applied to their development. The iPSC‐derived ECs show better junctional integrity, decreased inflammatory response, and increased flow‐responsive gene expression when they are grown on compliant nanofibrous matrices than standard culture systems [94]. The delivery of stem cells and extracellular vesicles through biomaterials results in better local retention of the stem cells while protecting the cargo from degradation and permitting controlled release of the stem cells into damaged arteries. The advanced models demonstrate that hybrid scaffolds combine VEGF‐mimetic peptides, notch‐activating ligands, and mechanosensitive polymers. Researchers developed these materials to create scaffolds that support full re‐endothelialization while preventing restenosis [95].

Next‐generation scaffolds aim not merely to support engraftment but to reconstruct the arterial microenvironment, which includes proper shear stress detection, extracellular matrix structure, and immune‐vascular interactions. The platforms will transform from operating as vessel replacements to functioning as systems that control vascular equilibrium because this development represents a critical advancement in arterial regeneration technology [96].

### 7.3 Gene‐Edited and Preconditioned Stem Cells

CRISPR engineering system enables scientists to achieve exact control over stem cell modifications, which scientists use for repairing arteries. The targeted editing system allows researchers to modify pathways that control ECs' ability to withstand damage, their paracrine secretion capacity, and their ability to maintain metabolic health, which results in improved regenerative abilities within ischemic and inflammatory arterial regions [97]. The strategies require two methods that stabilize eNOS expression and block pro‐senescent transcriptional programs, while they use two additional methods to enhance notch and KLF2 signaling for maintaining arterial identity. The editing methods enable researchers to remove immunogenic features, which results in better development of universal donor stem‐cell products [97, 98].

Stem cells undergo reprogramming through preconditioning strategies that include hypoxic priming and cytokine pulsing, metabolic rewiring, and biomechanical conditioning to develop phenotypes that function best in high‐stress vascular environments. Hypoxia‐adapted MSCs and endothelial progenitors demonstrate significant improvements in survival rates while showing a decrease in inflammatory response and an increase in extracellular vesicle cargo, which specifically targets pathways that neutrophils and macrophages use to control arterial remodeling [98]. Mechanical preconditioning through cyclic stretch and shear stress establishes arterial graft integration and function at the time of transplantation. The three changes that work toward a single objective aim to create cells that maintain their ability to regenerate while controlling immune response and establishing permanent endothelial and smooth muscle cell repair mechanisms. As scalable manufacturing platforms develop their capabilities, gene‐edited stem‐cell systems that undergo preconditioning will emerge as key clinical solutions for restoring arterial stability while treating progressive vascular disease [98, 99].

## 8 Precision Medicine and Future Directions in Arterial Regeneration

### 8.1 Precision Stratification and Patient‐Specific Vascular Profiling

The process of arterial regeneration will require scientists to discover solutions that help them understand the different biological characteristics, inflammatory responses, and regenerative powers of individual patients (Figure 4). The precision medicine frameworks now use multi‐omics vascular profiling with genomics, epigenomics, plasma proteomics, and single‐cell transcriptomics to discover the unique causes of ED and abnormal smooth muscle cell phenotypes and harmful immune‐vascular interactions that affect each patient [100, 101]. The datasets display different arterial remodeling patterns that affect how stem cell‐derived factors are responded to through variations in sensitivity to pro‐resolving cytokines and EV cargo and metabolic reprogramming signals. The development of therapeutic algorithms requires the integration of personalized signatures, which help identify patients who will respond to cell‐derived therapies and determine their optimal treatment times while reducing unintended inflammation [102, 103].

### 8.2 Predictive Modeling and Adaptive Treatment Design

Machine learning models built from longitudinal vascular imaging, circulating biomarker kinetics, and computational hemodynamics provide probabilistic estimates of arterial healing trajectories. The predictive tools help clinicians to determine which patients have the highest risk for three specific conditions: fibrous cap thinning, accelerated neointimal proliferation, and unstable plaque progression [104]. The system uses predictive modeling to create adaptive treatment schedules that treat patients through stem cell‐based interventions and allow for multiple low‐dose treatments of extracellular vesicles or stem cells with altered cytokine patterns during critical remodeling periods. The study results support the adaptive designs because research shows that one‐time treatments do not create lasting blood vessel restoration, and multiple treatments with regenerative signals help vascular ECs organize correctly while they prevent smooth muscle cells from developing their synthetic behavior, and they maintain stability in arterial microstructure [52, 105].

### 8.3 Integration of Genetic, Epigenetic, and Immune Signatures

Advanced development of precision vascular immunology demonstrates that individual patient immune profiles determine how their arteries will heal after surgical procedures. Stem cell‐based therapies, especially MSC‐ and cardiac‐resident progenitor‐derived factors, work together with specific macrophage and T‐cell phenotypes that differ between patients [106]. Epigenetic changes in SMCs and ECs through DNA methylation of contractile genes and histone marks that control inflammation‐enhanced fields affect their ability to regenerate. Future protocols will utilize pre‐treatment immune and epigenetic scoring to assess the need for immunomodulatory priming and metabolic conditioning, and small molecules that will improve patient response to stem cell‐derived stimuli. The complete integration of this method enables arterial regenerative therapy to function as a precise oncology treatment that uses biomarker‐based methods for patient care [107].

### 8.4 Stem Cell‐Guided Vascular Repair in Precision Therapeutics

Precision dosing and delivery become central as cell‐free formulations begin to take the driver's seat as the new dawn in the world of stem cell therapy. The personalized EV formulations, which contain specific microRNAs, pro‐homeostatic lipids, and chromatin‐modifying proteins, will provide suitable treatment options for specific arterial defects, including impaired endothelial regeneration, excessive SMC proliferation, and chronic macrophage activation [108, 109]. The study uses precision‐engineered stem cells that combine CRISPR‐based genetic modifications with synthetic gene circuits and microenvironment‐responsive promoters to deliver therapeutic factors exclusively through high‐risk arterial segments. These strategies enhance the delivery of drugs to the body while minimizing their effects throughout the organism and directing healing signals to the areas where blood vessel reconstruction occurs, rather than to healthy blood vessels [110].

### 8.5 Digital Twins and Multi‐Scale Models of Arterial Healing

The emergence of “vascular digital twins” serves as computational models to replicate a patient's arterial system through their biomechanics, molecular signaling, and cellular phenotypes provides researchers with an unmatched research tool to evaluate regenerative methods through virtual testing [111]. Digital twins improve therapeutic design through their ability to simulate how EV dosing and gene‐edited cell products and biomaterial scaffolds affect endothelial shear responses and SMC phenotypic transitions. Most of these models are suitable for experimental testing of the drug and can speed up the translation into the clinic of regenerative technologies [112].

The next decade of arterial regeneration will converge on modular, multimodal precision platforms that combine: (i) cell‐free bioactive factors tuned to individual vascular phenotypes; (ii) biomaterial carriers designed for anatomically precise delivery; (iii) minimally invasive repeated‐dose regimens; and (iv) biomarker‐guided stratification to match therapy with pathology [113]. Regulatory frameworks will increasingly require the use of mechanism‐based biomarkers and potency assays for EV and gene‐edited cell products and standardized predictive modeling methods. The future of arterial regeneration will advance from its current practice of applying standard treatments to developing specific treatment methods, which will provide personalized care through their continuous evaluation of patient needs and use of targeted anatomical therapeutics, combined with stem cell research and advanced medical techniques [114, 115].

## 9 Conclusion

Regenerative strategies for arterial disease have advanced far beyond the early expectation that transplanted stem cells would directly repopulate damaged vasculature. Converging evidence now establishes that the most meaningful therapeutic effects arise from cell‐derived signals that modulate endothelial repair, smooth muscle cell plasticity, and inflammatory resolution. These paracrine mechanisms delivered through cytokines, growth factors, metabolites, and extracellular vesicles form the biological basis for next‐generation interventions designed to stabilize plaques, restrain maladaptive remodeling, and restore arterial homeostasis.

Progress in bioengineering has further reshaped the field, enabling controlled delivery of stem cell‐derived factors, improving retention in arterial tissues, and reducing systemic exposure. Engineered vesicles, biomaterial scaffolds, and gene‐edited or preconditioned stem cells now offer the precision, potency, and reproducibility required for translation. These technologies directly address earlier limitations of cell survival, inconsistent engraftment, and short‐lived efficacy, positioning engineered paracrine therapeutics as plausible clinical candidates for vascular repair.

Precision medicine will be central to realizing the therapeutic promise of these platforms. Multi‐omic profiling and high‐resolution vascular phenotyping reveal that arterial remodeling, plaque vulnerability, and regenerative capacity vary substantially between individuals. Integrating these signatures into treatment design, combined with computational modeling and adaptive dosing strategies, will enable cell‐derived products to be tailored to the specific biological context of each patient, thereby increasing the likelihood of durable benefits.

Advances across stem cell biology, vascular engineering, and precision therapeutics now converge toward a unified framework for arterial regeneration. The path forward requires rigorous mechanistic studies, standardized potency assays, and well‐designed clinical trials capable of testing repeated dosing and biomarker‐guided stratification. These efforts will shift the management of arterial disease from damage containment to targeted restoration, marking a substantive step toward true regenerative cardiovascular medicine.

## Author Contributions

Yi Song conceived the review. Yun‐Yu Ma and Shu‐Yao Zhu collected data and wrote the initial draft. Yi Song and Yun‐Yu Ma revised the draft. All authors approved for submission.

## Funding

Fuwai Yunnan Cardiovascular Hospital 2025YFKT‐PY‐01.

## Ethics Statement

The authors have nothing to report.

## Consent

The authors have nothing to report.

## Conflicts of Interest

The authors declare no conflicts of interest.

## Data Availability

All the data are available in the manuscript and any specific information can be acquired from the corresponding author upon special request.
